# Measuring self-efficacy and outcome expectancy in evidence-based practice: A systematic review on psychometric properties

**DOI:** 10.1016/j.ijnsa.2021.100024

**Published:** 2021-03-05

**Authors:** P.A. Hoegen, C.M.A. de Bot, M.A. Echteld, H. Vermeulen

**Affiliations:** a Avans University of Applied Science, Expertise Centre Caring Society, Breda, the Netherlands; b Radboud Institute for Health Sciences, Radboud University Medical Center, IQ Healthcare Nijmegen, the Netherlands; c Faculty of Health and Social Studies, Research Department of Emergency and Critical Care, HAN University of Applied Sciences, Nijmegen, the Netherlands

**Keywords:** Evidence-based practice, Evidence based nursing, Quality of healthcare, Selfefficacy, Outcome expectancy, Measurement, Nurses, Psychometrics, systematic review

## Abstract

**Background:**

Evidence-based practice has developed over the last 30 years as a tool for the best possible nursing care. Nevertheless, many nurses do not regularly participate in the evidence-based practice process. Barriers to participation include nurses’ self-perceived ability in successfully fulfilling evidence-based practice-related tasks (self-efficacy) and their expectations of the positive outcomes of such tasks (outcome expectancy). To evaluate progress and provide feedback to professionals, monitoring the levels of self-efficacy and outcome expectancy with validated instruments is desirable. A comprehensive overview of the psychometric properties of such instruments is lacking.

**Objectives:**

To determine the psychometric properties of instruments designed to measure nurses’ self-efficacy and outcome expectancy in evidence-based practice.

**Design and method:**

This systematic review was performed on studies reporting psychometric properties of instruments that measure self-efficacy and outcome expectancy in EBP. MEDLINE, EMBASE and CINAHL databases were searched up to March 2020. Studies that reported psychometric properties on eligible scales and studied nurses or other healthcare professionals were included. Psychometric properties included content validity, construct validity, reliability, and responsiveness. The COSMIN risk of bias checklist and criteria for good measurement properties were applied independently by two reviewers. This review is registered with PROSPERO (CRD42020183069).

**Results:**

Eleven scales measuring self-efficacy or a similar construct and one scale measuring outcome expectancy were identified. The vast majority of the research focused on nurses. Internal consistency and structural validity were the most frequently reported properties, though the recommended confirmative factor analysis to verify the structural validity was rarely performed correctly. In addition, most studies that reported on construct validity did not hypothesise on the expected strength or direction of an effect before the data analysis. Responsiveness was not typically reported or was incorrectly studied. The included articles showed a high quality of evidence for four scales on structural validity and internal consistency. The Self-Efficacy in Evidence-Based Practice Activities scale showed the best content validity and was accompanied by an Outcome Expectations of Evidence-Based Practice scale. Both scales met the COSMIN standards for construct validity with high-quality evidence.

**Conclusions:**

In light of the evidence, the Self-Efficacy in Evidence-Based Practice Activities scale is considered promising, and along with the accompanying Outcome Expectations of Evidence-Based Practice scale, appears capable of accurately measuring both self-efficacy and outcome expectancy. The use of these scales is recommended, and further research should be conducted on the responsiveness of the scales.

## Abstract (author highlights)

• Nurses do not regularly participate in EBP. Lower levels of self-efficacy and outcome expectancy in EBP are presumed barriers that hinder nurses’ participation in EBP.
• Education in EBP is more effective to change knowledge, skills, attitudes and behaviour through interactive, and with professional practice integrated activities.
• Self-efficacy is influenced, among other things, by gaining positive experiences with tasks and positive feedback from relevant third parties, for example through coaching and training in professional practice.
• Measurement scales that focus on self-efficacy in EBP are available, therefore instead of developing new instruments, psychometric evaluation of the existing scales is more efficient.
• Out of eleven scales measuring self-efficacy and one measuring outcome expectancy in EBP, the questionnaires by Chang and Crowe have the best properties with respect to content validity, structural validity, cross-cultural validity and hypothesis testing.
• Despite that self-efficacy and outcome expectancy are related concepts, most instruments only aim at self-efficacy.
• Psychometric properties are not always investigated or reported in the best way, and researchers appear to have differing viewpoints on them.

**Summary box**

**What is already known about this topic?**

Nurses do not regularly participate in EBP. Lower levels of self-efficacy and outcome expectancy in EBP are presumed barriers that hinder nurses’ participation in EBP.

Education in EBP is more effective to change knowledge, skills, attitudes and behaviour through interactive, and with professional practice integrated activities.

Self-efficacy is influenced, among other things, by gaining positive experiences with tasks and positive feedback from relevant third parties, for example through coaching and training in professional practice.

Measurement scales that focus on self-efficacy in EBP are available, therefore instead of developing new instruments, psychometric evaluation of the existing scales is more efficient.

**What this paper adds?**

Out of eleven scales measuring self-efficacy and one measuring outcome expectancy in EBP, the questionnaires by Chang and Crowe have the best properties with respect to content validity, structural validity, cross-cultural validity and hypothesis testing.

Despite that self-efficacy and outcome expectancy are related concepts, most instruments only aim at self-efficacy.

Psychometric properties are not always investigated or reported in the best way, and researchers appear to have differing viewpoints on them.

Alt-text: Unlabelled box

## 1 Background

The Institute of Medicine (IOM) has advocated for the broad implementation of evidence-based practice (EBP) in healthcare to enhance the quality and safety of care. Evidence-based practice aims to improve the quality of care for patients through integrating evidence from scientific research, professionals’ expertise and patients’ preferences and values (Dawes et al., 2005; IOM, 2009). The concept of EBP has become generally accepted in healthcare as a method for improving the quality of care (Bleich, 2011; Medicine, 2009). Nevertheless, the use of EBP is not commonplace among healthcare professionals. Ubbink et al. (2013) outline various barriers to the adoption of EBP, including a lack of time and access to research publications and a lack of authority or ability to change care procedures. Ajzen’s (1991) and Bandura’s (1997) behavioural theories seem to apply to the latter barriers. Also, (Nagy et al., 2001) and Chang and Levin (2014) have also pointed out that low levels of confidence, or self-efficacy (SE) and outcome-expectancy (OE) also hinder EBP. Currently, SE in EBP is still one of the factors that need attention to bring EBP to the point of providing care (Boswell et al., 2020). A recent systematic review gathered assessment tools that evaluate EBP-teaching in medicine (Kumaravel et al., 2020). Unfortunately, self-reporting tools were excluded from that review, and none of the included instruments addressed SE or OE in EBP.  This psychometric review of potentially useful instruments was conducted to identify the most suitable existing instrument to measure levels of SE and OE in EBP.

Bandura's social cognitive theory (1997) differentiates two concepts that affect people's likelihood of attempting tasks. The first is SE, which is defined as one's self-perceived ability to organise and execute a specific task (Bandura, 1997). Individuals with a higher SE towards a specific task are more likely to undertake it.  The EBP process involves, for example, searching in databases or assessing the risk of bias. The second concept of OE involves one's judgement of the likely result of their behaviour (Bandura, 1997). For example, when nurses feel that their expertise is of no importance in wound policy, they are less likely to share their expertise when wound policy is decided with patients and healthcare professionals.

Education is known to increase knowledge about EBP (Coomarasamy and Khan, 2004); however, clinically integrated educational strategies also enhance skills and impact on EBP-related behaviours (Coomarasamy and Khan, 2004). Monitoring outcomes, such as knowledge and behaviour, as well as levels of SE and OE, is desirable when evaluating progress and providing feedback to professionals. Monitoring these outcomes over an extended period is necessary to evaluate the long-term effect of implementation strategies or education.

Potential monitoring instruments should provide; insight into a professional's level of SE and OE in EBP, are able to detect change overtime, and facilitate an evaluation of the success of educational and implementation programmes on developing SE and OE. Preliminary searches showed that several  instruments that measure EBP-related constructs have been developed. Therefore, rather than developing new instruments, the use of measurement scales that utilise the most appropriate psychometric properties is preferred (de Vet et al., 2011). This review aims to determine the psychometric properties of instruments designed to measure nurses’ self-efficacy and outcome expectancy in evidence-based practice.

## 2 Methods

### 2.1 Protocol and registration

A systematic review was conducted using the Preferred Reporting Items for Systematic Reviews and Meta-Analysis (PRISMA) statement (Moher et al., 2010) and the COSMIN protocol for the systematic review of measurement properties (Prinsen et al., 2018). Although SE and OE in EBP refer to healthcare professionals, not patients, this study applied the COSMIN criteria for Patient Reported Outcome Measures in light of the fact that questionnaires that measure EBP-related SE and OE constitute self-reported measurements of how professionals feel in relation to their SE and OE. The protocol for this review was registered in PROSPERO (CRD42020183069).

### 2.2 Information sources and search strategy

Final searches for studies on the development and validation of instruments that measure EBP-related SE and/or OE were conducted March 2nd, 2020 on the MEDLINE (through PubMed), EMBASE and CINAHL databases. The search terms utilised were ‘evidence-based practice’, ‘self-efficacy’, ‘outcome-expectancy’ and their synonyms, similar terms, and abbreviations. To focus the search strategies on studies on psychometric properties, the COSMIN filter for psychometric properties (Mokkink et al., 2018) were used. Additional searches were conducted using the partial names and abbreviations of questionnaires found in the major search. The search terms and strategies are listed in appendix 1. No limitations on the publication date or language were applied in the search strategies. A librarian at the Avans University of Applied Science, was consulted to verify the comprehensiveness of the searches.

### 2.3 Eligibility criteria and study selection

The criteria for study inclusion were: (1) obtained in full text, (2) reporting the psychometric properties of instruments measuring EBP-related self-efficacy and/or outcome expectancy and (3) including nurses at any educational level or other healthcare professionals. COSMIN recommendations were followed, and studies that did not clearly report on measurement properties were excluded (Mokkink et al., 2018; Prinsen et al., 2018). The eligibility was evaluated by two independent authors (PH and CdB). After the first screening of titles and abstracts, selected titles were obtained and full texts were read, and again seen through by the eligibility criteria by two authors (PH and CdB). After both selection rounds, if there was any disagreement, a third author (ME or HV) was consulted.

### 2.4 Data extraction

Information was extracted from the included studies by the first author using data tables and was cross-checked by the second and third author (CdB AND ME). Data were extracted based on the following general characteristics: author (s), publication date, title, name and language of the studied instrument, the study population and the number of study participants. To support the appraisal of face-validity, the items from each instrument were matched to the five consecutive steps of the EBP-process; asking (formulating an answerable question), acquiring (searching for and finding of scientific sources), appraising (evaluating the source’s quality and applicability), applying (integrating findings in practice) and assessing (evaluating outcomes and process) (Dawes et al., 2005).

### 2.5 Quality assessment

The methodological quality of each study was independently assessed for risk of bias by two authors (PH and CdB) using the COSMIN checklist for studies on measurement properties (Mokkink et al., 2018). The checklist includes requirements for each measurement property, such as performing a confirmative factor analysis and a suitable sample size to investigate construct validity, and proof of stability of the participants on the measured construct when testing reliability (Mokkink et al., 2018). In the event that a disagreement was unresolved after consulting the COSMIN manual (Mokkink et al., 2018), a third author was consulted (ME or HV). In accordance with the COSMIN checklist, a four-point rating scale (e.g., very good, adequate, doubtful, or inadequate) was applied for each applicable item of the checklist on measurement properties (Mokkink et al., 2018). The lowest rating given to a measurement property signalled its overall quality, which is presented in table 2 as the methodological quality per measurement property, per included article. When a measurement property was not reported, we considered an assessment of the property as being inapplicable to that study.

For each measurement instrument, the quality of evidence was graded based on the modified GRADE approach, as described in the ‘COSMIN manual for systematic reviews of PROMs’ (Mokkink et al., 2018). Unlike the regular GRADE approach, which distinguishes in advance between high-level trials and observational research into low-quality levels, COSMIN assumes that the overall results per measuring instrument are reliable and high quality (Mokkink et al., 2018, p. 33). The rating for the quality of evidence is highly dependentdependent on the COSMIN risk of bias assessment. The ratings are downgraded by one or two levels when the risk of bias criteria point to concerns about the quality of the evidence.

### 2.6 Synthesis

To answer the research question, the aspects of content validity (face validity), construct validity (structural validity, and hypothesis testing), reliability (test-retest reliability, and internal consistency) and responsiveness as defined by the COSMIN initiative were focused on (Mokkink et al., 2018, 2018). Definitions of the measurement properties were followed, and the COSMIN criteria for good measurement properties were applied (Mokkink et al., 2018; Prinsen et al., 2018).

Content validity is highly valued within the COSMIN standards as a prerequisite for further psychometric research (Mokkink et al., 2018). Whether the subscales matched either the constructs of EBP-related SE or OE and their comprehensiveness in relation to the EBP-process were investigated to examine face validity as a facet of content validity. In addition, notice was taken of two important aids for developing SE instruments, as described by Bandura, 2006. Firstly, items that measure SE should be formulated in a way that assesses capability rather than the degree of knowledge or understanding or views on utility (Bandura, 2006). Secondly, in terms of the response scale, Bandura, 2006 recommends a range from 0% to 100%, with 10% intervals or a numeric 0%–100% rating scale.

Structural validity refers to the extent to which scores reflect the dimensionality of the constructs measured (Mokkink et al., 2018). A confirmatory factor analysis (CFA) should be used to investigate structural validity. Criteria hereof are a comparative fit index (CFI) or Tucker-Lewis index (TLI) value higher than 0.95, the root mean square error of approximation (RMSEA) is lower than 0.06, or the standardised root mean residues (SRMR) is lower than 0.08 (Prinsen et al., 2018).

Internal consistency shows the degree of interrelatedness of the items of a measurement instrument or subscale (Prinsen et al., 2018) and is an aspect of reliability. Internal consistency is sufficient when there is at least some degree of evidence for structural validity and a Cronbach's alpha ≥ 0.70 for the subscales.

Test–retest reliability reflects whether a questionnaire is consistent over time and can identify whether the occurrence of variance is due to real differences between the measurements (Mokkink et al., 2018). Multiple measurements using one instrument with the same participants should result in similar scores when SE is unlikely to have changed in the time between the measurements. Continuous scores, such as the 0% to 100% scale, are studied by calculating an intraclass correlation coefficient (ICC). For ordinal scores, such as an 11-point rating scale, a kappa or weighted kappa is calculated. A value ≥ 0.7 for ICC or weighted Kappa is accepted (Prinsen et al., 2018).

Assuming that the instruments provide valid measurements, hypothesis testing is used to determine whether scores are consistent with predefined assumptions about the magnitude and direction of changes (Prinsen et al., 2018). For the hypothesis testing in the present study, generic hypotheses, as formulated by De Vet et al. (de Vet, Terwee, Mokkink, and Knol, 2011; Prinsen et al., 2018), were applied when no hypothesis had been formulated by the authors of an included study. Constructs that are related to SE, but not precisely the same (e.g., knowledge about EBP) should correlate between >0.30 and <0.50. Hypothesis testing can also determine whether an instrument measures a distinction between groups. Then, no effect would be expected when comparing similar groups and at least small effect-sizes when groups distinct on educational levels or before and after training in EBP.

## 3 Results

### 3.1 Study selection

**Fig. 1:**
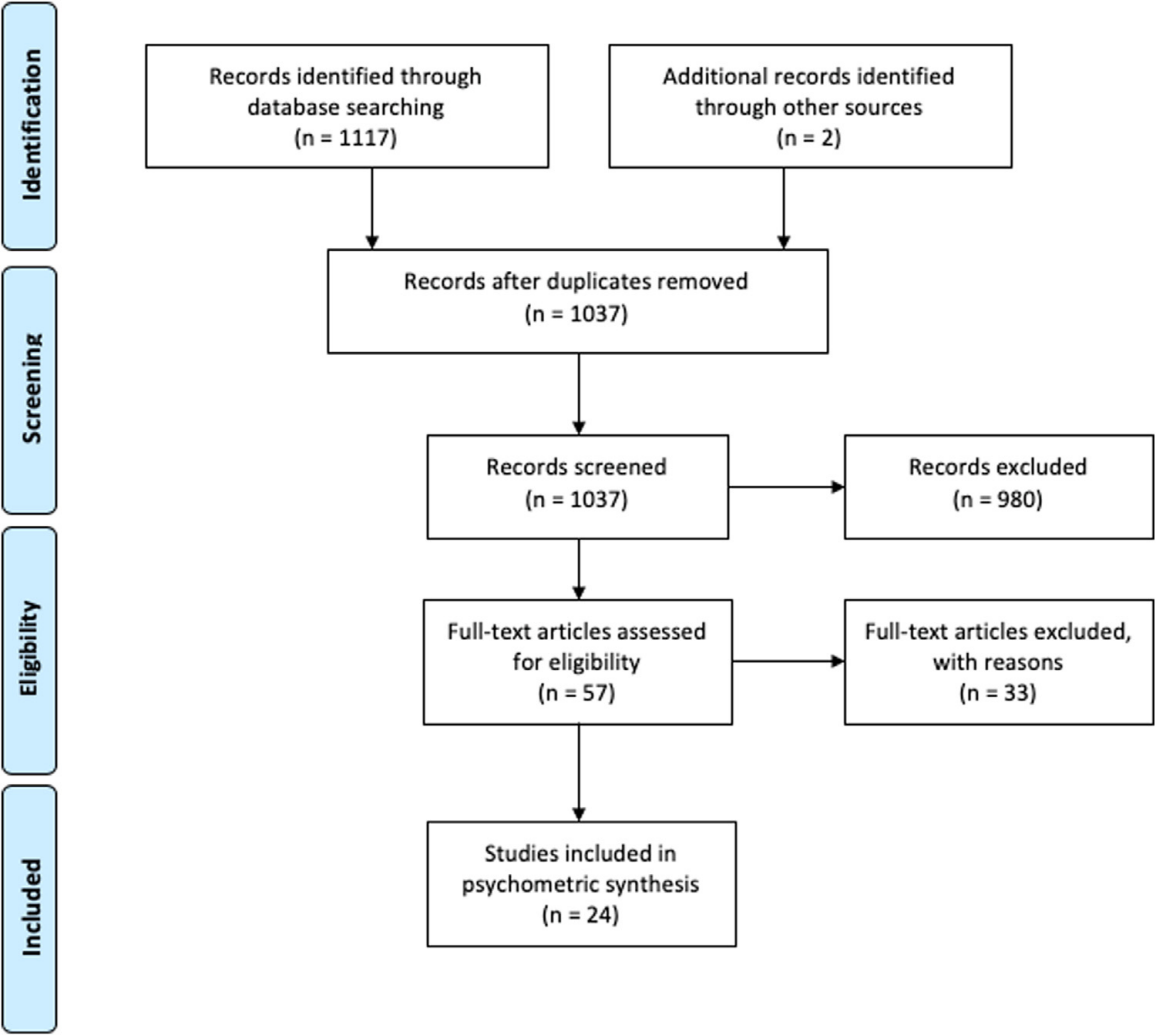
PRISMA Flow diagram of the selection process.

The search strategy identified 1117 studies. After cross-checking references and removing duplicates, 1037 articles were screened for eligibility. Twenty-four studies were subsequently included, as summarised in the flow diagram in Fig. 1.

### 3.2 Instrument and participants’ characteristics

A summary of the characteristics of the included studies is provided in Table 1. The 24 studies detailed 11 different instruments. Nine instruments were initially in English. The Evidence-Based Practice Beliefs (EBP Beliefs) scale (Melnyk et al., 2008) appeared translated and psychometrically studied in six other languages (Skela-Savič et al., 2017; Thorsteinsson, 2012; Verloo et al., 2017; Wang et al., 2012; Zelenikova et al., 2016). The Evidence-Based Practice Profile questionnaire (EBP2) (McEvoy et al., 2010) has been translated into and studied in Norwegian (Titlestad et al., 2017) and Polish (Belowska et al., 2018; Panczyk et al., 2017). Of the Self-efficacy in EBP (SE-EBP) and Outcome Expectancy for EBP instruments (OE-EBP) (Chang and Crowe, 2011; Ramis et al., 2019), only the SE-EBP instrument had been translated into Korean (Oh et al., 2016) . The other eight instruments had only been studied in their original language: the Evidence-Based Practice Attitudes, Self-Efficacy & Behavioural Implementation (EBP-At-SE-BI) (Watters et al., 2016), the Swedish EBP Capability Beliefs (EBP-CB) (Wallin et al., 2012), the Dutch EBP Self-efficacy and task value (EBP-SE/TV) (Spek et al., 2013), the EBP Survey (EBP-Survey) (Blackman and Giles, 2015), the EBP Self-efficacy (EBPSE) (Tucker et al., 2009), the Evidence-Based Practice Confidence (EPIC) (Clyde et al., 2016; Doble et al., 2018; Salbach and Jaglal, 2011; Salbach et al., 2013), the Knowledge, Attitudes, Access and Confidence Evaluation (KACE) (Hendricson et al., 2011) and the Nursing Research Self-Efficacy Scale (NURSES) (Swenson-Britt and Berndt, 2013).

**Table 1:** Summary of characteristics of the included studies and scales.

Reference	Construct	Scale	Status	Country / Language	Population	Availability	Number of items / scale type
McEvoy et al., 2010	Confidence^1^	EBP2	Original	Australia / English	Nursing and midwifery students	Yes, items in Belowska et al. (2018)	11 item subscale / 5-point scale
Titlestad et al., 2017	Confidence^1^	EBP2	Translation	Norway / Norwegian	Nursing students, social workers, social educator students, healty and social workers	Yes, items in Belowska et al. (2018)	11 item subscale / 5-point scale
Panczyk et al., 2017	Confidence^1^	EBP2	Translation	Poland / Polish	Nurses, midwives, and nursing and midwifery students	Yes, items in Belowska et al. (2018)	11 item subscale / 5-point scale
Belowska et al., 2018	Confidence^1^	EBP2	Translation	Poland / Polish	Nurses	Yes, items in appendix	11 item subscale / 5-point scale
Watters et al., 2016	SE^1^	EBP-At-SE-BI	Original	USA / English	Nursing students	Yes, items in article	9 items subscale / 4-point scale
Melnyk et al., 2008	Beliefs	EBP-Beliefs	Original	USA / English	Nurses	Yes, items in article	16 items subscale / 5-point scale
Wang et al., 2012	Beliefs (SE)	EBP-Beliefs	Translation	China / Chinese	Nurses	Yes, items in Melnyk et al. (2008)	16 items subscale / 5-point scale
Thorsteinsson, 2012	Beliefs	EBP-Beliefs	Translation	Iceland / Icelandic	Nurses	Yes, items in Melnyk et al. (2008)	16 items subscale / 5-point scale
Zelenikova et al., 2016	Beliefs	EBP-Beliefs	Translation	Czech Republic / Czech	Nursing students	Yes, items in Melnyk et al. (2008)	16 items subscale / 5-point scale
Zelenikova et al., 2016	Beliefs	EBP-Beliefs	Translation	Slovakia / Slovak	Nursing students	Yes, items in Melnyk et al. (2008)	16 items subscale / 5-point scale
Verloo et al., 2017	Beliefs	EBP-Beliefs	Translation	Switzerland / French	Nurses and allied healthcare providers	Yes, items in Melnyk et al. (2008)	16 items subscale / 5-point scale
Skela-Savič et al., 2017	Beliefs	EBP-Beliefs	Translation	Slovenia / Slovenian	Nurses	Yes, items in Melnyk et al. (2008)	16 items subscale / 5-point scale
Wallin et al., 2012	Capability beliefs	EBP-CB	Original	Sweden / Swedish	Nurses	Yes, items in article	6 items / 4-point scale
Chang and Crowe, 2011	SE and OE	SE-EBP OE-EBP	Original	Australia / English	Nurses	Yes, original manual	28 SE and 8 OE-items subscales / 11-point Likert scale
Oh et al., 2016	SE	SE-EBP	Translation	Korea / Korean	Nurses	Yes, original manual	28 SE items subscale / 11-point Likert scale
Ramis et al., 2019	SE	SE-EBP OE-EBP	Original	Australia / English	Nurses	Yes, original manual	28 SE and 8 OE-items subscales / 11-point Likert scale
Spek et al., 2013	SE^1^	EBP-SE/TV	Original	The Netherlands / Dutch	Speech-language therapy students	Yes, items in article	9 items subscale / 7-point Likert scale
Blackman and Giles, 2015	SE	EBP-Survey	Original	Australia / English	Nursing students	Yes, items in article	27 items / 4-point scale
Tucker et al., 2009	SE	EBPSE	Original	USA / English	Nurses	Yes, items in article	17 items / 0-100% rating scale
Salbach and Jaglal, 2011	Confidence	EPIC	Original	Canada / English	Healthcare professionals	Yes, items in appendix	11 items / 0-100% rating scale
Salbach et al., 2013	Confidence	EPIC	Original	Canada / English	Physical therapists	Yes, items in Salbach and Jaglal (2011)	11 items / 0-100% rating scale
Clyde et al., 2016	Confidence	EPIC	Original	Canada / English	Occupational therapists	Yes, items in Salbach and Jaglal (2011)	11 items / 0-100% rating scale
Doble et al., 2018	Confidence	EPIC	Original	Australia / English	Speech pathology students	Yes, items in Salbach and Jaglal (2011)	11 items / 0-100% rating scale
Hendricson et al., 2011	Confidence^1^	KACE Scales	Original	USA / English	Dental students	No.	6 items subscale / 5-point scale
Swenson-Britt and Berndt, 2013	SE	NURSES	Original	USA / English	Nurses	Yes, items in article	38 items / 5-point Likert scale

Legend: ATT = Attitudes; SE = Self-Efficacy; BI = Behavioural Implementation; TV = Task Value; OE = Outcome Expectancy; EPIC = Evidence-based Practice Confidence Scale; KACE = Knowledge, Attitudes, Access, and Confidence Evaluation; NURSES = Nursing Research Self-Efficacy Scale; 1: This instrument beholds more constructs than shown and relevant for this study.

Nine of 11 of the instruments targeted nurses, nursing students or other healthcare professionals combined with nurses. Details of the reviewed studies and scales are provided in Table 1.

### 3.3 Quality assessment and risk of bias

Most studies reported on structural validity (Table 2, COSMIN Box 3) and internal consistency (Table 2, COSMIN Box 4). One study reported on measurement error (Table 2, COSMIN Box 7). Reported and indirect information about hypothesis testing was used for both the hypothesis testing (Table 2, COSMIN Box 9) and the responsiveness (Table 2, COSMIN Box 10). The results of the quality assessment are given in Table 2. The property ‘criterion validity’ (Table 2, COSMIN Box 8) was not included in the table because it is impossible to study without a ‘golden standard’.

**Table 2:** Results of quality assessment and measurement properties of the included studies.

		Box 3 Structural validity			Box 4 Internal consistency			Box 5 Cross-cultural validity / measurement invariance			Box 6 Reliability		
			Methodological Result			Methodological Result			Methodological Result			Methodological Result	
Reference	Scale	n	quality^1^	(rating)^2^^,^^3^	n	quality^1^	(rating)^2^^,^^4^	n	quality^1^	(rating)^2^	n	quality	(rating)^2^^,^^5^
McEvoy et al., 2010	EBP-2	526	Adequate	(?)	105	Very good	α = .93 (?)		—		105	Doubtful	ICC = .83 (+)
Titlestad et al., 2017	EBP-2	149	Very good	CFI =.69 RMSEA =.089 SRMR =.095 (-)	149	Very good	α =.94 (?)	149	Very good	(-)	53	Adequate	ICC = .76 (95% CI .62 - .85) (+)
Panczyk et al., 2017	EBP-2	1362	Adequate	(?)	1362	Very good	α =.94 (?)	1362	Adequate	(?)		—	
Belowska et al., 2018	EBP-2	427	Inadequate	(?)	427	Very good	α =.97 (?)	427	Inadequate	(?)		—	
Watters et al., 2016	EBP-At-SE-BI	348	Adequate	(?)	348	Very good	α =.86 (?)		—			—	
Melnyk et al., 2008	EBP-Beliefs	333	Adequate	(?)	330	Very good	α =.90 (?)		—			—	
Wang et al., 2012	EBP-Beliefs		—		361	Very good	α =.88 (?)		—			—	
Thorsteinsson et al., 2012	EBP-Beliefs	-	Inadequate	(?)	471	Very good	α =.86 (?)	471	Doubtful	(?)		—	
Zelenikova et al., 2016	EBP-Beliefs	132	Adequate	(?)	132	Very good	α =.85 (?)	132	Doubtful	(?)		—	
Zelenikova et al., 2016	EBP-Beliefs	91	Doubtful	(?)	91	Very good	α =.82 (?)	91	Doubtful	(?)		—	
Verloo et al., 2017	EBP-Beliefs	382	Adequate	(?)	382	Very good	α =.88 (?)	382	Doubtful	(?)		—	
Skela‐Savič et al., 2017	EBP-Beliefs	780	Adequate	(?)	760	Very good	α =.92 (?)	780	Adequate	(?)		—	
Wallin et al., 2012	EBP-CB (LANE)	1256	Adequate	(?)	1256	Inadequate	(?)		—			—	
Chang et al., 2011	EBP-SE/OE	165	Adequate	(?)	165	Very good	α =.97 (?)		—			—	
Oh et al., 2016	EBP-SE/OE	212	Very good	CFI =.91 TLI =.90 RMSEA =.08 (-)	212	Very good	α =.95 (?)	212	Very good	(+)		—	
Ramis et al., 2019	EBP-SE/OE	201	—	(?)	210	—	(?)		—			—	
Spek et al., 2013	EBP-SE/TV	149	Adequate	(?)	164	Very good	α =.79 (?)		—			—	
Blackman et al., 2015	EBP-Survey Scale	375	Adequate	(?)		Doubtful	(?)		—			—	
Tucker et al., 2009	EBPSE		Inadequate	(?)	93 / 80	Very good	α =.95α =.97 (?)		—			—	
Salbach et al., 2011	EPIC		—			—	(?)		—			—	
Salbach et al., 2013	EPIC	275	Adequate	(?)	275	Very good	α =.89 (?)		—		187	Doubtful	ICC =.89 (+)
Clyde et al., 2016	EPIC		—			—			—		79	Doubtful	ICC =.92 (+)
Doble et al., 2018	EPIC		—		159	Doubtful	α =.83 α =.88 (?)	159	Inadequate	(?)		—	
Hendricson et al., 2011	KACE Scales		—		151 92	Very good	α =.87 α =.94 (?)		—		70	Doubtful	(?)
Swenson-Britt et al., 2013	NURSES	649	Very good	CFI =.99 RMSEA =.063 SRMR =.0225 (+)	649	Very good	α =.983 (+)		—			—	

		Box 7 Measurement error			Box 9 Hypothesis testing			Box 10 Responsiveness		
			Methodological Result			Methodological Result			Methodological Result	
Reference	Scale	n	quality^1^	(rating)^2^^,^^6^	n	quality^1^	(rating)^2^	n	quality^1^	(rating)^2^
McEvoy et al., 2010	EBP-2		—		105	Adequate	(+)		—	
Titlestad et al., 2017	EBP-2	53	Adequate	SEM= .38 (?)	96	Very good	(+)	96	Very good	(+)
Panczyk et al., 2017	EBP-2		—		1362	Adequate	(-)		—	
Belowska et al., 2018	EBP-2		—		427	Adequate	(?)		—	
Watters et al., 2016	EBP-At-SE-BI		—		348	Doubtful	(+)	348	Inadequate	(?)
Melnyk et al., 2008	EBP-Beliefs		—		330	Adequate	(+)		—	
Wang et al., 2012	EBP-Beliefs		—		361	Adequate	(+)		—	
Thorsteinsson et al., 2012	EBP-Beliefs		—		471	Doubtful	(+)		—	
Zelenikova et al., 2016	EBP-Beliefs		—		132	Adequate	(+)		—	
Zelenikova et al., 2016	EBP-Beliefs		—		91	Adequate	(+)		—	
Verloo et al., 2017	EBP-Beliefs		—			—			—	
Skela‐Savič et al., 2017	EBP-Beliefs		—			—			—	
Wallin et al., 2012	EBP-CB (LANE)		—		1084	Adequate	(+)		—	
Chang et al., 2011	EBP-SE/OE		—		165	Adequate	(+)		—	
Oh et al., 2016	EBP-SE/OE		—		212	Adequate	(+)		—	
Ramis et al., 2019	EBP-SE/OE		—		210	Very good	(+)	210	—	
Spek et al., 2013	EBP-SE/TV		—		164	Adequate	(+)		—	
Blackman et al., 2015	EBP-Survey Scale		—			—			—	
Tucker et al., 2009	EBPSE		—		53 40	Adequate	(+)	53 40 30	Adequate	(+)
Salbach et al., 2011	EPIC		—			—			—	
Salbach et al., 2013	EPIC		—		275	Very good	(+)		—	
Clyde et al., 2016	EPIC		—		126	Adequate	(+)		—	
Doble et al., 2018	EPIC		—		159	Very good	(+)	159	Adequate	(+)
Hendricson et al., 2011	KACE Scales		—		231	Adequate	(-)	24	Adequate	(+)
Swenson-Britt et al., 2013	NURSES		—			—			—	

^1^ : — = no information available.

^2^ : (+) = sufficient; (-) = insufficient; (?) = indetermediate.

^3^: CFI = comparatice fit index; RMSEA = root mean square error of approximation; SRMR = standardised root mean square residual.

^4^ : α = Cronbachs alpha.

^5^ : ICC = intraclass correlation coefficient; CI = confidence interval.

^6^ : SEM = standard error of measurement.

The quality of evidence for 11 scales measuring SE or a similar construct and one subscale measuring OE was subsequently assessed. Studies that included professionals other than nurses were downgraded in terms of the quality of evidence. One study comprised a study sample of smaller than 100 and was subsequently downgraded by one level. As most scales were only reported in one study, they were not downgraded for inconsistent results of the measurement scales. The results are shown in Table 3.

**Table 3:** Quality of evidence per measurement scale.

Scale	N of studies		GRADE quality of evidence (modified GRADE approach (Mokkink et al., 2018))					
		Box 3 Structural validity	Box 4 Internal consistency	Box 5 Cross-cultural validity	Box 6 Reliability	Box 7 Measurement error	Box 9 Hypothesis testing	Box 10 Responsiveness
**EBP^2^**	4	⊗⊗⊗⊗ High	⊗⊗⊗⊗⊗ High	⊗⊗⊗⊗ High	⊗⊗⊗○ Moderate	⊗⊗⊗○ Moderate	⊗⊗⊗⊗ High	⊗⊗⊗⊗ High
**EBP-At-SE-BI**	1	⊗⊗⊗○ Moderate	⊗⊗⊗⊗ High	–	–	–	⊗⊗○○ Low	⊗○○○ Very low
**EBP-Beliefs**	7	⊗⊗⊗⊗ High	⊗⊗⊗⊗ High	⊗⊗○○ Low	–	–	⊗⊗⊗⊗ High	–
**EBP-CB**	1	⊗⊗⊗○ Moderate	⊗○○○ Very low	–	–	–	⊗⊗⊗○ Moderate	–
**SE-EBP**	3	⊗⊗⊗⊗ High	⊗⊗⊗⊗ High	⊗⊗⊗⊗ High	–	–	⊗⊗⊗⊗ High	–
**OE-EBP**	2	⊗⊗⊗○ Moderate*	⊗⊗⊗⊗ High	–	–	–	⊗⊗⊗⊗ High	–
**EBP-SE/TV**	1	⊗⊗○○ Low	⊗⊗⊗○ Moderate	–	–	–	⊗⊗○○ Low	–
**EBP Survey**	1	⊗⊗⊗○ Moderate	⊗⊗⊗○○ Low	–	–	–	–	–
**EBPSE**	1	⊗○○○ Very low	⊗⊗⊗○ Moderate	–	–	–	–	–
**EPIC**	4	⊗⊗○○ Low	⊗⊗⊗○ Moderate	○○○○ -	⊗⊗○○ Low	–	⊗⊗⊗○ Moderate	⊗⊗⊗○ Moderate
**KACE**	1	–	⊗⊗⊗○ Moderate	–	⊗○○○ Very low	–	⊗⊗○○ Low	⊗○○○ Very low
**NURSES**	1	⊗⊗⊗⊗ High	⊗⊗⊗⊗ High	–	–	–	–	–

### 3.4 Validity

The scales were compared to the steps of the EBP process (Table 4) to assess the content validity. This revealed that the KACE Scales (Hendricson et al., 2011) only covered the third step relating to evidence appraisal. The EBP2 (McEvoy et al., 2010), EBP-At-SE-BI (Watters et al., 2016), EBP-Beliefs (Melnyk et al., 2008), EBP-SE/TV (Spek et al., 2013), EBP-Survey (Blackman and Giles, 2015) and NURSES (Swenson-Britt and Berndt, 2013) also omitted certain EBP steps. Four scales covered all five steps of the EBP process: the EBP-CB (Wallin et al., 2012), SE-EBP (Chang and Crowe, 2011), EBPSE (Tucker et al., 2009) and the EPIC scale (Clyde et al., 2016; Doble et al., 2018; Salbach and Jaglal, 2011; Salbach et al., 2013).

**Table 4:** Items representing steps in the EBP process per measurement scale.

Scale		Steps of the EBP process^1^					
	N of items	Step 1 Ask	Step 2 Acquire	Step 3 Appraise	Step 4 Apply	Step 5 Assess (evaluate)	Other items
EBP^2^	11	(2) 34, 35,	(3) 36, 37, 38,	(3) 39, 40, 41,	(1) 42,	(0)	(2) 32, 33,
EBP-At-SE-BI	9	(1) C4,	(2) C1, C8,	(4) C2, C5, C6, C7,	(0)	(0)	(2) C3, C9,
EBP-Beliefs	16	(0)	(1) 6,	(0)	(3) 7, 14, 15	(1) 10,	(11) 1, 2, 3, 4, 5, 8, 9, 11, 12, 13, 16
EBP-CB	6	(1) 1,	(2) 2, 3,	(1) 4,	(1) 5,	(1) 6,	(0)
SE-EBP	28	(5) 1, 2, 3, 4, 5,	(8) 6, 7, 8, 9, 10, 11, 12, 13,	(7) 14, 15, 16, 17, 18, 19, 20,	(4) 21, 22, 23, 24,	(5) 25, 26, 27, 28	(0)
OE-EBP	8	(1) 1,	(4) 2, 3, 4, 5	(0)	(2) 6, 7	(1) 8	(0)
EBP-SE/TV	9	(0)	(2) 2, 3	(2) 4, 7	(0)	(0)	(5) 1, 5, 6, 8, 9,
EBP-Survey	27	(2) 3, 8,	(1) 1,	(7) 5, 6, 13, 18, 19, 21, 27	(5) 2, 15, 16, 17, 20,	(0)	(12) 4, 7, 9, 10, 11, 12, 14, 22, 23, 24, 25, 26
EBPSE	17	(1) 1,	(4) 2, 3, 4, 5,	(3) 4, 5, 11,	(9) 6, 7, 8, 9, 10, 12, 13, 14, 17,	(1) 15,	(1) 16,
EPIC	11	(2) 1, 2	(1) 3,	(5) 4, 5, 6, 7, 8,	(2) 9, 10,	(1) 11,	(0)
KACE	6	(0)	(0)	(6)	(0)	(0)	(0)
NURSES	39	(1) 24	(6) 1 - 6	(13) 7 - 19	(1) 27	(0)	(17) 20 - 23, 25, 26, 28 - 39

1 : The number in brackets is the number of items of the (sub) scale that concern this step in the EBP process.

2 : The numbers without brackets refer to the item number on the relevant (sub) scale.

To further assess the content validity, the studies were checked to determine whether Bandura's (Bandura, 2006) advice on developing measurement instruments had been followed. The scales SE-EBP (Chang and Crowe, 2011) and EBPSE (Tucker et al., 2009a) were found to have been formulated in accordance with Bandura's (Bandura, 2006) recommendations as a judgement of capability. The SE-EBP, OE-EBP (Chang and Crowe, 2011), EBPSE (Tucker et al., 2009a) and EPIC (Salbach and Jaglal, 2011) also used the recommended response scales.

To test the structural validity, a CFA was applied by Wang et al. (2012), Oh et al. (2016) and Swenson-Britt & Berndt (2013). The NURSES scale was the only scale that met the COSMIN criteria (Prinsen et al., 2018). The other scales were not studied with CFA; for this reason, no reference values are given in Table 2.

In terms of the cross-cultural validity, none of the included studies performed a multi-group confirmative factor analysis (MGCFA), regression analysis or differential item functioning (DIF) analyses with data collected from the original and translated questionnaires. Two studies performed a CFA based on the factor structure of the original questionnaire (Oh et al., 2016; Titlestad et al., 2017) and were rated ‘very good’. One study (Swenson-Britt and Berndt, 2013) performed a CFA using data from the original questionnaire and was, therefore, deemed ‘not applicable’ for the purposes of the cross-cultural validity.

Four scales were supported with high quality evidence for hypothesis testing. Most of the tested and accepted hypothesis referred to known-groups validity, where discriminative validity was tested between two or more groups of people who should score differently on the outcome, based on different characteristics such as educational levels.

### 3.5 Reliability

All reported Cronbach's alpha values were above the cut-off value of 0.70; however, because sufficient structural validity was conditional for internal consistency (Prinsen et al., 2018), most studies were rated indeterminate (Table 2).

The quality of evidence for the reliability for the EBP2-scale was rated as ‘moderate’ and had accepted ICCs of over 0.70. The EPIC scale also met this cut-off value but had a low quality of evidence. No ICC or weighted Kappa was reported for the KACE scale, which resulted in a low-quality and indeterminate rating.

### 3.6 Responsiveness

Responsiveness based on hypothesis testing was studied for four scales. One scale was found to have high-quality evidence (Table 3). One study (Watters et al., 2016) performed a before–after study but changed the measurement instrument between the two measures. Therefore, it not clear whether values that changed did so due to genuine change or whether the change was partly due to the revised scale.

## 4 Discussion

This review sought to determine the measurement properties of instruments measuring self-efficacy (SE) and outcome expectancy (OE) in EBP among nurses. Eleven scales measuring SE or a similar construct and one scale measuring OE were identified following a comprehensive search. The included articles showed high-quality evidence for structural validity, and internal consistency for four of the measurement scales found (Table 3). Of these scales, Chang and Crowe's (2011) SE-EBP held the best content validity. The SE-EBP covered all five steps of the EBP process, and followed Bandura's (Bandura, 2006) recommendations on the formulation of items and the response scale. With the exception of criterion validity, which was not studied for any scale, all properties were known of the EBP2 scale (McEvoy et al., 2010). This scale also demonstrated high-quality evidence and confirmed the hypothesis testing and responsiveness (Titlestad et al., 2017). In addition, the SE-EBP (Chang and Crowe, 2011) met the COSMIN standards for hypothesis testing with high-quality evidence (Chang and Crowe, 2011; Oh et al., 2016; Ramis et al., 2019).

The well-accepted Consensus Based Standards for the selection of health Measurement Instruments (COSMIN) criteria (Prinsen et al., 2018) were applied to conduct this psychometric review. Helpfully, one of the authors (PH) had participated in a three-day course organised by members of the COSMIN workgroup on the interpretation of these guidelines. The included articles were identified in this study through a comprehensive broad search, supplemented with specific searches for articles on the identified instruments.  The applied search strategy contained search strings specifically aimed at psychometric studies and studies on the development of measuring instruments. As a result, some studies that contain implicit information about psychometrics may not have been found until the specific, hand search. An independent quality assessment, data extraction and a thorough discussion of the findings also ensured the validity and reliability of the conclusions drawn.

None of the studies included reported data on all measurement properties. Internal consistency and structural validity were most often studied. Some methodological issues were identified following an evaluation of the studies.

Firstly, in relation to structural validity, CFAs were rarely performed correctly. Exploratory factor analyses were applied but did not deliver statistics for model fit. Only one of the three studies that did perform a CFA met the cut-off values imposed by COSMIN (Mokkink et al., 2018); however, this particular study performed an exploratory factor analysis and a subsequent CFA on the same dataset, which is not recommended by COSMIN. The two studies that did not match the COSMIN standards used translated instruments.

Secondly, when assessing construct validity, it was found that most studies did not provide any hypotheses on the expected strength or direction of a difference or correlation before the data analysis. When comparing known groups, the p-value was often reported, which reflects the chance of a difference or correlation deviating from zero difference or no correlation. The p-value does not provide information on the validity of a difference or correlation between measures; therefore, it is not relevant to construct validity (de Vet et al., 2011).

Lastly, it is likely that instruments that measure SE and OE in EBP are used to detect changes in response to courses, training and other implementation activities that are designed to impact on SE and/or OE. Therefore, responsiveness should be studied when content validity, structural validity and internal consistency are accepted. Studying responsiveness requires a longitudinal design where some participants within a closed cohort are very likely to change on the construct measured (de Vet et al., 2011). Therefore, comparing two different groups of professionals or students in different stages of training does not reflect responsiveness but construct validity through hypothesis testing (de Vet et al., 2011).

Quality appraisal is highly dependent on the completeness and clarity of the included studies. In addition, because this study investigated nurses’ SE and OE in EBP, it was necessary to downgrade the strength of evidence for studies that only included other professionals as participants because measurement properties relevant to one profession may not apply to others. As a result, the quality of evidence assessments may vary slightly when conducted for other healthcare professionals.

This review provides an overview of the currently available instruments for measuring SE and OE in EBP and also assesses their measurement properties. Following a review of potential suitable instruments measuring solely SE and OE, the SE-EBP and OE-EBP scales (Chang and Crowe, 2011) were shown to be the most suitable on the basis of their content validity and subsequently appraised quality of evidence. However, the SE-EBP scale is lengthy with 28 items. Future research may seek to reduce the number of items in this scale, while keeping content validity in mind.

## 5 Conclusions

This study identified 11 self-reported questionnaires on SE in EBP and one subscale on OE in EBP. The SE-EBP and OE-EBP scales (Chang and Crowe, 2011) were shown to be the best-suited scales for translation and use in practice.

The studies included in this review did not provide insight into all the measurement properties of each scale. This was due to the studies’ authors’ different views on psychometric research methods and their purposes, as well as shortcomings in reporting the results. However, the information gathered supports the preference for translating and using existing instruments as opposed to developing new ones (de Vet et al., 2011). Future research that utilises the questionnaires referenced in this study should seek to report all the possible measurement properties to build a thorough psychometric base for those instruments.

Content validity is considered a key requirement, followed by structural validity and internal consistency. The SE-EBP and OE-EBP questionnaires by Chang and Crowe (2011) were found to have the most favourable characteristics and measurement properties. In light of the evidence, further psychometric research that investigates cross-cultural validation and responsiveness with the use of the SE-EBP and OE-EBP scales is recommend.

## Declaration of Competing Interest

None.

## Appendix Supplementary materials

Image, application 1
