# Supplementary material for: Measuring self-efficacy and outcome expectancy in evidence-based practice: A systematic review on psychometric properties
Source: Int J Nurs Stud Adv. 2021 Mar 5;3:100024. doi: 10.1016/j.ijnsa.2021.100024 (PMC11080366; doi:10.1016/j.ijnsa.2021.100024)
Supplement: Supplementary file 1 [file mmc1.docx]

PUBMED

| ID | Search |
| --- | --- |
| #2 | (scale[tiab] OR scales[tiab] OR measure[tiab] OR measurement[tiab] OR measuring[tiab] OR measures[tiab] OR tool[tiab] OR tools[tiab] OR instrument[tiab] OR instruments[tiab]) |
| #3 | (validation[tiab] OR validity[tiab] OR reliability[tiab] OR developing[tiab] OR development[tiab] OR creation[tiab] OR adaption[tiab] OR adaptation[tiab] OR psychometric[tiab] OR measurement properties[tiab]) |
| #4 | Self Efficacy[Mesh] OR self efficacy[tiab] OR confident[tiab] OR confidence[tiab] OR faith[tiab] OR trust[tiab] OR beliefs[tiab] OR capability beliefs[tiab] OR (capabilities[tiab] AND beliefs[tiab]) OR outcome expectancy[tiab] OR outcome expectancies[tiab] OR outcome expectation[tiab] OR outcome expectations[tiab] |
| #5 | Evidence-Based Nursing[Mesh] OR Evidence-Based Practice[Mesh] OR evidence based practice[tiab] OR evidence based[tiab] OR EBP[tiab] OR EBN[tiab] |
| #6 | #2 AND #4 AND #5 |
| #7 | #2 AND #3 AND #4 AND #5 |

| #8 | ((instrumentation[sh] OR methods[sh] OR "Validation Study"[pt] OR "Comparative Study"[pt] OR "psychometrics"[MeSH] OR  psychometr*[tiab] OR clinimetr*[tw] OR clinometr*[tw] OR "outcome assessment, health care"[MeSH] OR "outcome assessment"[tiab] OR "outcome measure*"[tw] OR "observer variation"[MeSH] OR "observer variation"[tiab] OR "Health Status Indicators"[Mesh] OR "reproducibility of results"[MeSH] OR reproducib*[tiab] OR "discriminant analysis"[MeSH] OR reliab*[tiab] OR unreliab*[tiab] OR valid*[tiab] OR "coefficient of variation"[tiab] OR coefficient[tiab] OR homogeneity[tiab] OR homogeneous[tiab] OR "internal consistency"[tiab] OR (cronbach*[tiab] AND (alpha[tiab] OR alphas[tiab])) OR (item[tiab] AND (correlation*[tiab] OR selection*[tiab] OR reduction*[tiab])) OR agreement[tw] OR precision[tw] OR imprecision[tw] OR "precise values"[tw] OR test-retest[tiab] OR (test[tiab] AND retest[tiab]) OR (reliab*[tiab] AND (test[tiab] OR retest[tiab])) OR stability[tiab] OR interrater[tiab] OR inter-rater[tiab] OR intrarater[tiab] OR intra-rater[tiab] OR intertester[tiab] OR inter-tester[tiab] OR intratester[tiab] OR intra-tester[tiab] OR interobserver[tiab] OR inter-observer[tiab] OR intraobserver[tiab] OR intra-observer[tiab] OR intertechnician[tiab] OR intertechnician[tiab] OR intratechnician[tiab] OR intra-technician[tiab] OR interexaminer[tiab] OR inter-examiner[tiab] OR intraexaminer[tiab] OR intra-examiner[tiab] OR interassay[tiab] OR inter-assay[tiab] OR intraassay[tiab] OR intra-assay[tiab] OR interindividual[tiab] OR inter-individual[tiab] OR intraindividual[tiab] OR intra-individual[tiab] OR interparticipant[tiab] OR inter-participant[tiab] OR intraparticipant[tiab] OR intra-participant[tiab] OR kappa[tiab] OR kappa's[tiab] OR kappas[tiab] OR repeatab*[tw] OR ((replicab*[tw] OR repeated[tw]) AND (measure[tw] OR measures[tw] OR findings[tw] OR result[tw] OR results[tw] OR test[tw] OR tests[tw])) OR generaliza*[tiab] OR generalisa*[tiab] OR concordance[tiab] OR (intraclass[tiab] AND correlation*[tiab]) OR discriminative[tiab] OR "known group"[tiab] OR "factor analysis"[tiab] OR "factor analyses"[tiab] OR "factor structure"[tiab] OR "factor structures"[tiab] OR dimension*[tiab] OR subscale*[tiab] OR (multitrait[tiab] AND scaling[tiab] AND (analysis[tiab] OR analyses[tiab])) OR "item discriminant"[tiab] OR "interscale correlation*"[tiab] OR error[tiab] OR errors[tiab] OR "individual variability"[tiab] OR "interval variability"[tiab] OR "rate variability"[tiab] OR (variability[tiab] AND (analysis[tiab] OR values[tiab])) OR (uncertainty[tiab] AND (measurement[tiab] OR measuring[tiab])) OR "standard error of measurement"[tiab] OR sensitiv*[tiab] OR responsive*[tiab] OR (limit[tiab] AND detection[tiab]) OR "minimal detectable concentration"[tiab] OR interpretab*[tiab] OR ((minimal[tiab] OR minimally[tiab] OR clinical[tiab] OR clinically[tiab]) AND (important[tiab] OR significant[tiab] OR detectable[tiab]) AND (change[tiab] OR difference[tiab])) OR (small*[tiab] AND (real[tiab] OR detectable[tiab]) AND (change[tiab] OR difference[tiab])) OR "meaningful change"[tiab] OR "ceiling effect"[tiab] OR "floor effect"[tiab] OR "Item response model"[tiab] OR IRT[tiab] OR Rasch[tiab] OR "Differential item functioning"[tiab] OR DIF[tiab] OR "computer adaptive testing"[tiab] OR "item bank"[tiab] OR "cross-cultural equivalence"[tiab]))  NOT  ("address"[Publication Type] OR "biography"[Publication Type] OR "case reports"[Publication Type] OR "comment"[Publication Type] OR. "directory"[Publication Type] OR "editorial"[Publication Type] OR "festschrift"[Publication Type] OR "interview"[Publication Type] OR "lecture"[Publication Type] OR "legal case"[Publication Type] OR "legislation"[Publication Type] OR "letter"[Publication Type] OR "news"[Publication Type] OR "newspaper article"[Publication Type] OR "patient education handout"[Publication Type] OR "popular work"[Publication Type] OR "congress"[Publication Type] OR "consensus development conference"[Publication Type] OR "consensus development conference, nih"[Publication Type] OR "practice guideline"[Publication Type]) |
| --- | --- |
| #9 | #2 AND #4 AND #5 AND #8 |

EMBASE

| ID | Search |
| --- | --- |
| #2 | self efficacy.mp. or (self efficacy or confident or confidence or faith or trust or beliefs or capability beliefs or outcome expectancy or outcome expectancies OR outcome expectation or outcome expectations).ti,ab. or (capability and beliefs).ti,ab. |
| #3 | (evidence based or evidence based practice or EBP or EBN).ti,ab. |
| #4 | (scale or scales or measure or measurement or measuring or measures or tool or instrument or instruments) and (validation or validity or reliability or developing or development or creation or adaption or adaptation or psychometric or measurement properties)).ti,ab. |
| #5 | #2 AND #3 AND #4 |
| #6 | #1 AND #2 AND #3 AND #4 |
| #7 | #5 excluding MEDLINE |

CINAHL

| ID | Search |
| --- | --- |
| #2 | MH “self efficacy” OR TI ( self efficacy OR confident OR confidence OR faith OR trust OR beliefs OR capability beliefs ) OR TI ( capabilities AND beliefs ) OR AB ( self efficacy OR confident OR confidence OR faith OR trust OR beliefs OR capability beliefs ) OR AB ( capabilities AND beliefs ) OR TI (  outcome expectancy OR outcome expectancies OR outcome expectation OR outcome expectations) OR AB (  outcome expectancy OR outcome expectancies OR outcome expectation OR outcome expectations) |
| #3 | (MM "Nursing Practice, Evidence-Based+") OR (MH "Professional Practice, Evidence-Based+") OR "Evidence-Based" ) OR TI ( "Evidence-Based" OR EBN OR EBP ) OR AB ( "Evidence-Based" OR EBN OR EBP ) |
| #4 | TI (scale or scales or measure or measurement or measuring or measures or tool or instrument or instruments) OR AB (scale or scales or measure or measurement or measuring or measures or tool or instrument or instruments) |
| #5 | TI ( validation or validity or reliability or developing or development or creation or adaption or adaptation or psychometric or measurement properties ) OR AB ( validation or validity or reliability or developing or development or creation or adaption or adaptation or psychometric or measurement properties ) |
| #6 | #4 AND #5 |
| #7 | #2 AND #3 AND #4 AND #5 |
| #8 | #7 excluding MEDLINE |
